# Supplementary material for: Crawling and Gliding: A Computational Model for Shape-Driven Cell Migration
Source: PLoS Comput Biol. 2015 Oct 21;11(10):e1004280. doi: 10.1371/journal.pcbi.1004280 (PMC4619082; doi:10.1371/journal.pcbi.1004280)
Supplement: S1 Code — (ZIP) [file pcbi.1004280.s012.zip › release/tst/doc/html/globals_vars.html]

Tissue Simulation Toolkit: File Members


Tissue Simulation Toolkit
 0.1.4.1


- Main Page
- Namespaces
- Classes
- Files

- File List
- File Members

- All
- Functions
- Variables
- Typedefs
- Macros

- copyprob
  : ca.cpp
- errno
  : x11graph.cpp
- par
  : ca.cpp
  , cell.cpp
  , cell.h
  , dish.cpp
  , info.cpp
  , parameter.cpp
  , pde.cpp
  , x11graph.cpp
- Quiet
  : warning.cpp


---

Generated on Thu Aug 14 2014 22:04:02 for Tissue Simulation Toolkit by  

 1.8.6
